# The TREAT-NMD care and trial site registry: an online registry to facilitate clinical research for neuromuscular diseases

**DOI:** 10.1186/1750-1172-8-171

**Published:** 2013-10-23

**Authors:** Sunil Rodger, Hanns Lochmüller, Adrian Tassoni, Kathrin Gramsch, Kirsten König, Kate Bushby, Volker Straub, Rudolf Korinthenberg, Janbernd Kirschner

**Affiliations:** 1 Institute of Genetic Medicine, Newcastle University, International Centre for Life, Central Parkway, Newcastle upon Tyne NE1 3BZ, UK; 2 Clinical Trials Unit, University Medical Center Freiburg, Elsässer Strasse, 2, 79110, Freiburg, Germany; 3 Department of Neuropaediatrics and Muscle Disorders, University Medical Center Freiburg, Mathildenstrasse 1, 79106, Freiburg, Germany

**Keywords:** Rare diseases, Registries, Clinical trials, Trial sites, Care sites, Centres of expertise, CTSR, Reference networks, ERN, CARE-NMD

## Abstract

**Background:**

Rare diseases pose many research challenges specific to their scarcity. Advances in potential therapies have made it more important than ever to be able to adequately identify not only patients with particular genotypes (via patient registries) but also the medical professionals who provide care for them at particular specialist centres of expertise and who may be competent to participate in trials. Work within the neuromuscular field provides an example of how this may be achieved.

**Methods:**

This paper describes the development of the TREAT-NMD Care and Trial Site Registry (CTSR), an initiative of an EU-funded Network of Excellence, and its utility in providing an infrastructure for clinical trial feasibility, recruitment, and other studies.

**Results:**

285 CTSR-registered centres, reporting 35,495 neuromuscular patients, are described alongside an analysis of their provision for DMD. Site characteristics vary by country: the average number of DMD patients seen per site in the United States (96) is more than in Germany (25), and paediatric/adult breakdown is also markedly distinct. Over 70% of sites have previous trial experience, with a majority including a Clinical Trials Unit. Most sites also have MLPA diagnostic capability and access to a range of medical specialists. However, in the three countries reporting most sites (US, the UK and Germany), few had access to all core DMD specialists internally. Over 60% of sites did not report any form of transition arrangement.

**Conclusions:**

Registries of care and trial sites have significant utility for research into rare conditions such as neuromuscular diseases, demonstrated by the significant engagement by industry and other researchers with the CTSR. We suggest that this approach may be applicable to other fields needing to identify centres of expertise with the potential to carry out clinical research and engage in clinical trials. Such registries also lend themselves to the developing context of European Reference Networks (ERNs), which seek to build networks of centres of expertise which fit specific criteria, and which may themselves aid the sustainability of such registries. This is particularly the case given the utility of registries such as the CTSR in enabling networks of best-practice care centres.

## Background

Rare diseases, those affecting fewer than 5 in 10,000 EU citizens, pose a range of challenges to researchers [1]. Identifying sufficient patients with a given condition, let alone a specific genotype, to conduct meaningful clinical trials, natural history studies or health care research is difficult in small populations. Limited patient cohorts have been a barrier to the development of new therapies for rare diseases. Orphan drugs legislation, designed to encourage investment in the field, has had a positive impact with an unprecedented number of clinical trials initiated for rare diseases. However, it has also exposed a lack of trial readiness in many rare disease communities, with an increasing need to identify both patients who might participate in studies and sites with sufficient experience to take part in trials.

The requirement to improve trial readiness for the neuromuscular field was addressed via infrastructures developed by the EU FP6-funded TREAT-NMD network of excellence. Within this network, a series of patient registries, frequently established and run in collaboration with patient organisations, have established a resource for the identification of patients with specific genotypes and diagnoses (Duchenne Muscular Dystrophy, Spinal Muscular Atrophy and other neuromuscular diseases) [2]. Other disease areas have taken a similar approach with the most recent Orphanet Report “Disease Registries in Europe” cataloguing 588 registries [3,4].

Patient identification is merely part of the challenge of establishing clinical trials in rare diseases. Only a limited number of clinics exist with experience in caring for patients with rare diseases and conducting research in the field, and those for any specific disease are often few and far between. Such issues have spurred the development and activities of specialised professional networks for a variety of rare and more common conditions, including the Parkinson’s Study Group, the European Cystic Fibrosis Society, and the European Huntington’s Disease Network [5-7].

Neuromuscular diseases form a group of conditions often treated by the same professionals. Specialist neuromuscular clinics caring for patients with Duchenne muscular dystrophy (DMD) will often also see patients with Spinal Muscular Atrophy (SMA), Congenital Muscular Dystrophies (CMDs) and sometimes other conditions such as Charcot-Marie-Tooth disease (CMT) and Limb-Girdle Muscular Dystrophies (LGMDs) [8]. Those working in such clinics are thus likely to possess specialist skills and expertise with applicability to a range of neuromuscular conditions. However, identifying the locations of such sites and their capabilities has hitherto proved difficult.

We describe the development and utilisation of an online registry of care and trial sites for neuromuscular disorders that addresses these challenges, the TREAT-NMD Care and Trial Site Registry (CTSR), operational since December 2007.

## Methods

The CTSR is an online self-registration database for neuromuscular centres hosted by the University Medical Center Freiburg, Germany. Its initial aim was to enable clinical trial feasibility enquiries, but it has since been used for a range of feasibility, trial, and research enquiries.

The CTSR technical platform comprises a Java web application running on a MySQL database accessed via a secure web server. This allows swift self-registration and update of information by any internet-connected centre, regardless of geographic location. The application is designed in such a way to permit future extension.

CTSR questions were chosen via expert consensus within the TREAT-NMD network, and are based on typical industry feasibility enquiries and EUCERD recommendations [9]. They are wide-ranging across four broad categories: Patient Cohort, Care Settings, Research and Education, and Clinical Trial Infrastructure (Table 1). Further detail is available on the TREAT-NMD website [2].

**Table 1:** Simplified description of data captured in TREAT-NMD CTSR

**Patient cohort**	Patients stratified by disease and age range (currently 10 NMDs including subtypes e.g. SMA I, II, III).
	Diagnostic tools as most appropriate for each condition.
**Care settings**	Availability of specialists and services in-centre.
	Arrangements for transition care.
	Availability of particular pulmonary, cardiac, muscle and bone function tests in-centre.
	Availability of particular physiotherapy facilities and equipment in-centre.
	Availability of emergency care in-centre.
	Experience of centre in conducting skeletal muscle biopsies.
**Research and education**	Extent of use of centre data in research, research funding arrangements, and papers authored by staff at centre
	Extent to which staff at centre have been involved in providing training at national and international levels
**Clinical trial infrastructure**	Available personnel (e.g. Study Nurses, Physiotherapists, Pharmacists)
	Previous experience (e.g. details of past participation in Phase I, II, III, IV clinical trials)
	Availability and details of equipment (e.g. refrigerators, IT support)

Two additional sections capture project-specific data: a questionnaire on DMD care provision, used by the CARE-NMD project to investigate care for DMD across 7 European countries; and a series of qualitative questions for UK neuromuscular sites developed in conjunction with the Muscular Dystrophy Campaign [10,11].

Centres register for the CTSR via the TREAT-NMD website. All requests are manually curated to ensure the validity of new registrants. A user agreement must be signed and registrants can define the purposes for which their data may be used. Once registered, data is entered into online forms organised by the topic categories indicated above. Sites are recruited globally by a variety of means, particularly via professional networks, including TREAT-NMD and its associated communications infrastructure, such as the TREAT-NMD newsletter which is distributed monthly to 3,500 recipients. The CTSR is also frequently publicised at conferences and meetings, and through societies with a neuromuscular focus. In a country-specific approach, accredited neuromuscular centres were approached individually through key contacts where available.

As part of the user agreement, sites are asked to indicate whether their data might be forwarded to third parties [12]. A typical enquiry is initiated by an approach of an academic institution or a private company to the TREAT-NMD Secretariat at Newcastle University. A preliminary form records broad enquiry parameters, including timelines, target disease and genotypic criteria. In some cases, the enquiry may utilise the TREAT-NMD Global Database (an international federated database of National Patient Registries) in addition to the CTSR. Once an enquiry goes ahead, the CTSR team generates the dataset defined by the terms of the enquiry, and creates a report containing the requested information from all sites that have agreed to forward their data. After each enquiry all sites are informed about the enquiry and the data that has been provided. The requesting party receives relevant information to answer their enquiry, but does not get uncontrolled access to the full CTSR database.

## Results

### Sites and patient cohort

**Figure 1:**
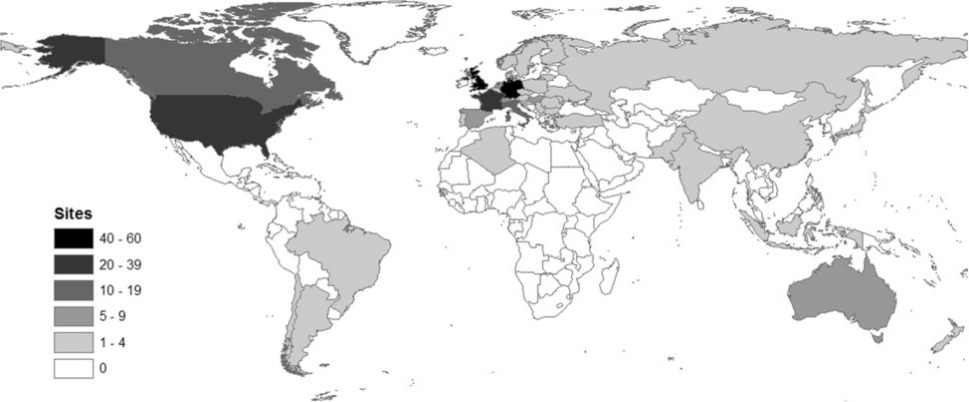
Map showing number of sites registered in the CTSR by country.

285 centres in 42 countries are registered in the CTSR, distributed according to Figure 1. These are characterised by country in Table 2. The three countries with the largest number of registered centres are Germany (60), the UK (41), and the United States (38), totalling 48.8% of sites. This reflects the initial focus of TREAT-NMD on Europe with close US collaboration, though the presence of registered sites worldwide (including in Japan, Australia, and South America) also demonstrates the evolution of the network into the TREAT-NMD Alliance with a global governance structure.

**Table 2:** CTSR site distribution by country, by total number of patients per country

**Country**	**Sites**	**Total NMD patients**	**Avg # patients per site**	**Total DMD patients**
United States	38	7495	197	2777
United Kingdom	41	5765	141	1514
Italy	19	4408	232	795
Germany	60	3909	65	1127
France	20	2206	110	373
Denmark	3	1356	452	186
Canada	10	1008	101	458
Belgium	3	875	292	219
Japan	3	784	261	230
Australia	8	764	96	426
Russia	4	737	184	206
Netherlands	6	691	115	152
Poland	4	628	157	166
Turkey	3	620	207	215
Spain	6	529	88	102
Countries reporting 100–500 NM patients*	36	3130	36	1171
Countries reporting <100 NM patients**	21	590	21	270
Totals	285	35495	125	10387

*Austria, Brazil, Bulgaria, China, Czech Republic, Hungary, India, Israel, Serbia, Sweden, Switzerland, Ukraine.

**Algeria, Argentina, Chile, Croatia, Finland, Indonesia, Lithuania, Macedonia, Moldova, New Zealand, Norway, Pakistan, Portugal, Romania, Slovenia.

The 285 centres reported a total of 35,495 patients across 10 diseases (distinguishing between SMA Type I, Type II, and Type III) with the most commonly reported being DMD (10,387 patients, 29% of overall cohort) followed by Myotonic Dystrophy Type I (DM1, 6,043 patients, 17% of overall cohort) and SMA (5,062 patients, 14% of overall cohort). A breakdown of the CTSR cohort by disease and paediatric/adult status is provided in Table 3.

**Table 3:** Patient cohort by disease and age

	**<18**	**18+**	**Total**
**DMD**	8184	2203	10387
**BMD**	1301	1570	2871
*Subtotal B/DMD*	*9485*	*3773*	*13258*
**SMA I**	758	42	800
**SMA II**	1989	487	2476
**SMA III**	890	896	1786
*Subtotal SMA*	*3637*	*1425*	*5062*
**LGMD**	1630	3798	5428
**CMD**	918	221	1139
**CM**	947	418	1365
**FSHD**	586	2614	3200
**DM1**	1325	4718	6043
*Subtotal Other*	*5406*	*11769*	*17175*
**Total**	**18528**	**16967**	**35495**

**Figure 2:**
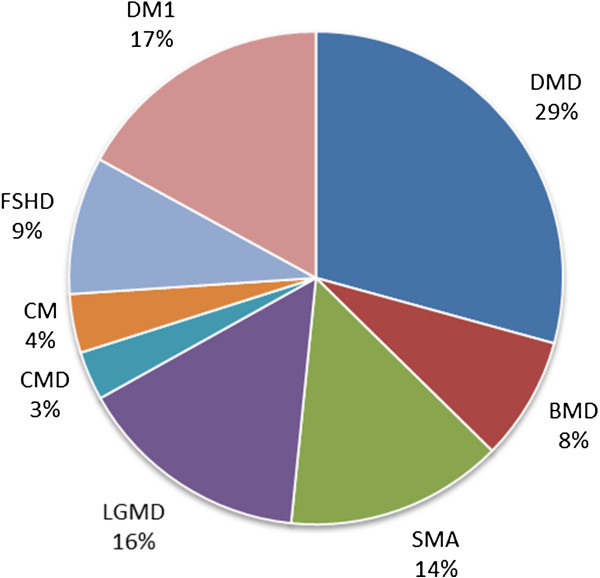
Overall patient cohort of CTSR.

**Figure 3:**
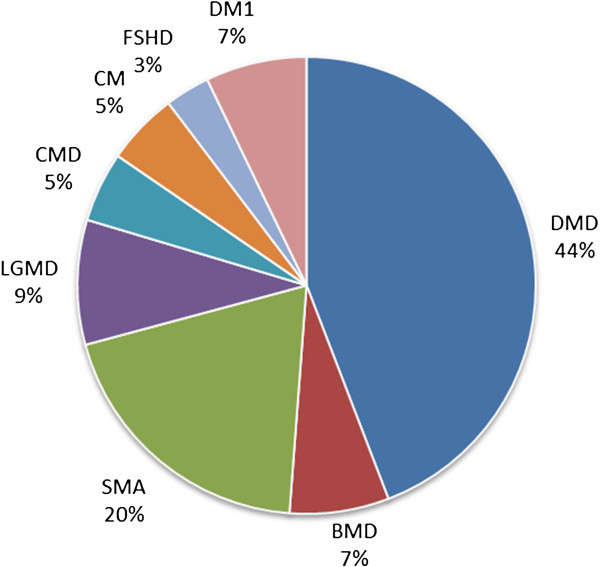
Paediatric patient cohort of CTSR.

**Figure 4:**
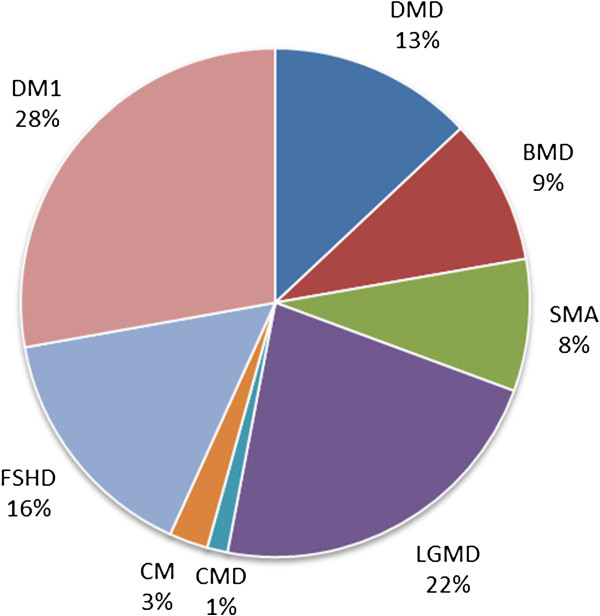
Adult patient cohort of CTSR.

Figures 2, 3, and 4 show the relative numbers of patients with different neuromuscular conditions in the CTSR. The prominence of DMD, particularly in the paediatric cohort, is expected as this disease is the most common form of childhood muscular dystrophy. In the adult cohort, the increased prominence of primarily adult-onset conditions (particularly Myotonic Dystrophy, Facioscapulohumeral Muscular Dystrophy, and Limb-Girdle Muscular Dystrophies) is apparent. We focus primarily on DMD in the following analysis.

Table 4 provides details of the demographics of the DMD population represented in the CTSR. The three countries with the largest number of DMD patients in the CTSR – the United States, United Kingdom and Germany – are the same as those with the largest number of sites in the CTSR, and the proportion of adult to paediatric DMD patients is broadly similar (27.6%, 24.8% and 27.1% respectively). Sites in these three countries report 5,418 DMD patients, about half (52.2%) of the overall DMD cohort (4,277 of 8,184 or 52.3% of adults; 1,141 of 2,203 or 51.8% of children).

**Table 4:** Demographics of DMD population represented in the CTSR

**Country**	**Sites reporting DMD patients**	**DMD patients <18**	**DMD patients 18+**	**Total DMD patients**	**Avg <18 pats/DMD site**	**Avg 18+ pats/DMD site**	**Avg pats/DMD site**	**Sites reporting only DMD Pats <18**	**Sites reporting only DMD Pats 18+**	**Sites reporting patients both <18 and 18+**
United States	29	2177	600	2777	75	21	96	1	0	28
United Kingdom	29	1213	301	1514	42	10	52	8	7	14
Germany	46	887	240	1127	19	5	25	22	7	17
Italy	15	557	238	795	37	16	53	4	0	11
Canada	9	387	71	458	43	8	51	1	0	8
Australia	3	407	19	426	136	6	142	1	0	2
France	14	159	214	373	11	15	27	1	7	6
Countries reporting between 100–250 DMD patients*	41	1798	442	2240	61	20	81	15	2	24
Countries reporting less than 100 DMD patients**	22	599	78	677	27	3	30	10	0	12
**Total**	**208**	**8184**	**2203**	**10387**	**39**	**9**	**48**	**63**	**23**	**122**

*Belgium, Czech Republic, Denmark, Hungary, India, Israel, Japan, Netherlands, Poland, Russia, Spain, Switzerland, Turkey.

**Austria, Brazil, Bulgaria, Chile, China, Croatia, Finland, Indonesia, Moldova, Norway, Pakistan, Portugal, Romania, Serbia, Slovenia, Sweden, Ukraine.

Differences are apparent between countries in the size of individual centres, particularly between those focusing on adults and paediatric patients. Considering only those specialist centres that report seeing DMD patients, the average number of DMD patients at each such centre in the United States (96) is almost double that in the UK (52), which in turn is double that in Germany (25). Disparities in centre size are noted in other countries too: the average number of DMD patients in France (27) is comparable to that Germany, while Italy (53) and Canada (51) are more similar to the UK.

There are also differences with respect to breakdown and provision of paediatric and adult care for DMD. In the United States, 28 of 29 centres report treating both paediatric and adult patients: only one is a purely paediatric centre for DMD and none are specialist adult centres. Many of these may be paediatric centres who continue to provide care to patients as adults. This is in contrast to the UK and Germany, where sites are split more evenly between those seeing only paediatric, only adult, or both paediatric and adult patients. Further characterisation of the sites in each country is provided in Table 5. This shows the characteristics of registered neuromuscular sites treating DMD patients, ordered by the average size of each site in each country. It is clear that in those countries where sites are fairly large (significantly more than 50 DMD patients per site on average), there are relatively few sites in each country. The exception to this is the United States, where there are a both a large number of sites and a large average DMD population per site.

**Table 5:** Characteristics of CTSR sites seeing DMD patients

**Country**	**Sites reporting DMD patients**	**Total DMD patients**	**Minimum number of DMD patients per site**	**Maximum number of DMD patients per site**	**Avg pats/DMD site**
Japan	1	230	230	230	230
Turkey	1	215	215	215	215
Australia	3	426	51	205	142
Israel	1	109	109	109	109
United States	29	2777	12	398	96
India	2	183	77	106	92
Pakistan	1	80	80	80	80
Belgium	3	219	33	104	73
Russia	3	206	44	95	69
Denmark	3	186	10	160	62
Czech Republic	3	163	10	110	54
Italy	15	795	8	187	53
United Kingdom	29	1514	3	252	52
Canada	9	458	18	155	51
Austria	1	50	50	50	50
Countries reporting a mean of 20–49 DMD patients per site*	91	2594	1	208	29
Countries reporting a mean of <20 DMD patients per site**	13	182	44	96	14

*Brazil, Bulgaria, China, France, Germany, Hungary, Moldova, Netherlands, Norway, Poland, Portugal, Romania, Serbia, Spain, Sweden.

**Chile, Croatia, Finland, Indonesia, Slovenia, Switzerland, Ukraine.

Table 6 provides characteristics of “adult-only” sites that see DMD patients in the three countries where there is more than one such site: the United Kingdom, Germany and France (seven such sites in each country). The average number of patients seen at these adult-only sites is low: 8, 3, and 18 respectively, which is much lower than the average number of DMD patients seen at all sites in each country (29, 46 and 24). Again, one observes striking differences in character of neuromuscular sites between different countries.

**Table 6:** Characteristics of sites seeing only adult DMD patients (in countries with >1 such site)

**Country**	**Sites reporting DMD patients**	**Sites reporting ONLY DMD pats <18**	**Sites reporting ONLY DMD pats 18+**	**Sites reporting patients both <18 and 18+**	**Avg patients per site seeing only 18+**	**Avg pats/DMD site**
United Kingdom	29	8	7	14	8	52
Germany	46	22	7	17	3	25
France	14	1	7	6	18	27

### Utility

**Figure 5:**
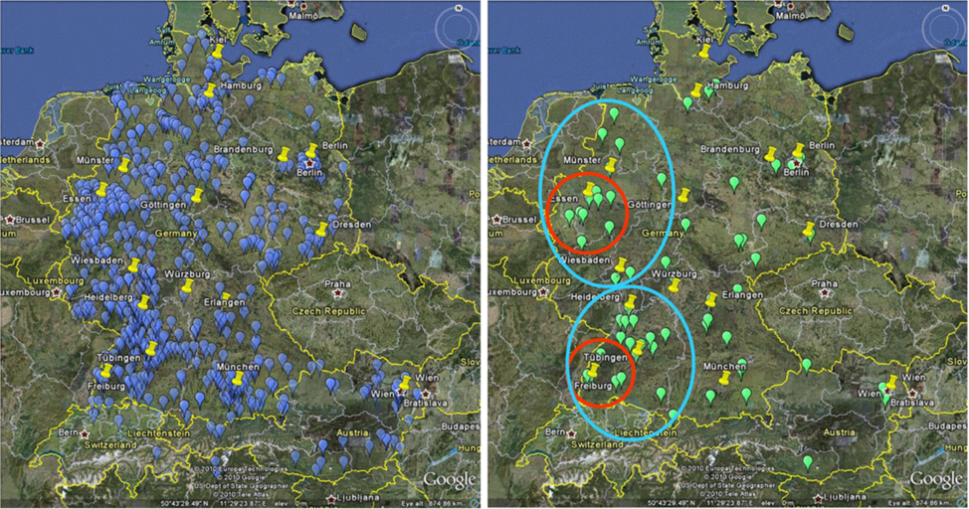
**Maps demonstrating utility of combined CTSR and National Patient Registry data for recruitment.** Left, map combining data from German/Austrian Patient Registries and CTSR to show location of DMD patients (blue dots) in relation to CTSR sites (yellow dots). Right, map showing only Exon 51-skippable DMD patients (green dots) under the care of two CTSR sites (red circles) and extended recruitment area for clinical trial (within 2 hours travel time, blue circles).

CTSR data on neuromuscular sites and their patient cohort helps to plan clinical trials in small populations. A CTSR enquiry – particularly when combined with an enquiry of the relevant National Patient Registries, as shown in Figure 5 (data from 2012) – permits the identification of the most effective locations for clinical trial centres via clusters of patients and sites with particular attributes.

### Site attributes

The CTSR holds data on a range of site attributes, which have wide applicability and utility to researchers. These include questions related to clinical trial experience and capabilities, which enables the identification of sites with particular expertise and equipment. Questions in the CTSR were developed mindful of the wider context of evolving criteria for the designation of Centres of Expertise for Rare Diseases in European Member States as published by EUCERD [9]. A discussion of a selection of key site attributes is provided below.

### Clinical research and trials

#### Trial and good clinical practice experience

Table 7 shows several variables associated with the experience of centres in conducting clinical trials. Answers to these questions are not restricted to neuromuscular trials, but could cover all clinical trial experience.

**Table 7:** Trial and GCP experience

**Trial and good clinical practice**								
	**Familiarity with good clinical practice**	**Availability of a clinical trials unit**	**Previous trial experience**	**Trial phase I experience**	**Trial phase II experience**	**Trial phase III experience**	**Trial phase IV experience**	**Currently participating in a trial**
Yes	188	124	177	49	124	112	62	144
No	56	112	68	236	161	173	223	101

77.0% of reporting sites are familiar with Good Clinical Practice, whilst just over half (52.5%) have a Clinical Trials Unit available. Overall, the majority of reporting sites (72.2%) have some previous clinical trial experience. Of those providing a more detailed breakdown of their experience, relatively few have taken part in Phase I trials (17.2%) and Phase IV trials (21.8%), while more have taken part in Phase II (43.5%) and Phase III (39.3%) trials. This is most likely because relatively few sites are required for Phase I trials, while Phase II/III trials often take place at the same centres. In the neuromuscular field, few drugs have received marketing authorisation and the number of Phase IV trials is still relatively low, but will require many sites across different countries to reach their recruitment targets.

### Care settings

#### Diagnostic techniques

Swift and accurate diagnosis is a very important aspect of good care for neuromuscular conditions, and enables genetic counselling for the family. For DMD, Multiplex ligation-dependent probe amplification (MLPA) is the minimum capability recommended in international consensus care standards [13]. This is available and funded in 149 (70.3%) of 212 reporting sites, with the more advanced ability to detect point mutations available and funded at 137 (64.6%) of these sites (Table 8).

**Table 8:** Techniques available for the diagnosis of DMD and SMA*

**Availability of specific diagnostic techniques**							
	**DMD**				**SMA**		
	**Limited deletion**	**MLPA**	**Point mutation**	**Muscle biopsy**	**SMA deletion**	**Point mutation**	**Copy number**
Available, funded	176	149	137	173	163	116	98
Available, not funded	35	42	51	37	37	40	52
Not available	13	21	33	13	8	40	42

*Availability includes in-house as well as external genetic testing.

#### Availability of medical specialists

Neuromuscular diseases often require the expertise of a team of medical specialists with a range of expertise. The nature of such a multidisciplinary team, co-ordinated by an expert in the disease, is described as applied to DMD by Bushby *et al.*[13]. The majority of sites have core specialities available internally, although this availability varies depending on speciality (Tables 9 and 10).

**Table 9:** Availability of specific medical specialists for neuromuscular patients

**Availability of specific medical specialists**					
	**Neurologist**	**Pulmonologist**	**Cardiologist**	**Genetic counselling**	**Physiotherapist**
Internal/team member/joint clinics	125	93	82	79	117
External	4	35	46	48	11

**Table 10:** Availability of core specialists in countries with the largest number of sites in the CTSR

**Availability of specific medical specialists**					
	**Neurologist**	**Pulmonologist**	**Cardiologist**	**Genetic counselling**	**Physiotherapist**
Internal/team member/joint clinics	125	93	82	79	117
External	4	35	46	48	11

In the three countries with the most sites in the CTSR, relatively few sites reporting DMD patients had all core specialists available internally, via a team member, or via joint clinics: 15 of 46 (32.6%) in Germany, 8 of 29 (27.6%) in the UK, and 5 of 29 in the US (17.2%). Where this is the case, many instead refer to relevant local provision, serving to organise care which is delivered by other providers: 40 of 46 (87.0%) in Germany, 22 of 29 (75.9%) in the UK, and 12 of 29 in the US (41.4%). Again, differences in availability of specialists highlight differences in care organisation between countries.

#### Transition care

For conditions with childhood onset such as DMD, transition from paediatric to adult care can be challenging, but it is also crucial as more children with the condition are living into adulthood. Table 11 shows the transition arrangements of sites in the CTSR. It is notable that a majority (62.8%) of sites do not have any form of transitional arrangement, a finding which lends support to patient experiences described in other studies such as that by Abbot *et al.*[14].

**Table 11:** Transition arrangements for adult care summary table

**Adult care arrangement**	**Number of sites**
No transitional arrangement	179
Joint clinic (pediatric and adult neurologist)	54
Regular personal contact between pediatric and adult neurologist	52

### Examples of use of the CTSR

The CTSR had been used for eight industry enquiries by May 2013 (Table 12), as well as four academic enquiries.

**Table 12:** Industry enquiries to the CTSR (PR: Patient Registries)

**Company**	**Date**	**Type**	**Disease**	**Registry**	**Geographic coverage**
Prosensa	July 2009	Feasibility enquiry	DMD	CTSR & PR	Europe, USA, Canada
Acceleron	September 2009	Feasibility enquiry	FSHD	CTSR only	Worldwide
Trophos	March 2010	Feasibility enquiry	SMA	CTSR & PR	Europe
Acceleron	June 2010	Feasibility enquiry	DMD	CTSR & PR	Europe, USA, Canada, Japan
Santhera	December 2010	Feasibility enquiry	DMD	CTSR & PR	Europe, USA, Canada
Company X	November 2011	Feasibility enquiry (refinement and update)	DMD	CTSR only	Worldwide
Ultragenyx	November 2012	Feasibility enquiry	GNE myopathy	CTSR only	Worldwide
Eli Lilly	May 2013	Feasibility enquiry	DMD	CTSR & PR	Worldwide

### Feasibility enquiry

Industry and academic enquiries were used to support drug development plans and to preselect potential trial sites for clinical research. The combined use of patient registry and CTSR data often proved especially helpful to assess feasibility of clinical trials in rare NMDs. For the FOR-DMD steroid trial and other investigator initiated trials the CTSR was used to collect relevant feasibility information from all participating sites.

### CARE-NMD

CARE-NMD, a three-year EU funded project to improve access to best-practice care for DMD across 7 EU countries (Bulgaria, the Czech Republic, Denmark, Germany, Hungary, Poland and the United Kingdom) used the CTSR in several capacities [11]. These included the identification of neuromuscular sites, distribution of professional questionnaires, and the supplementation of existing knowledge from national networks such as the North Star Clinical Network in the UK [15]. The project demonstrated the flexibility of this web-based platform, extending the CTSR with additional data fields. A series of questions relating to care provision for DMD were developed based on published international consensus care standards, and integrated with the CTSR [13,16].

In the United Kingdom parallel initiatives were underway to improve knowledge of the state of neuromuscular care. These included audits of care provision by the Muscular Dystrophy Campaign and the British Myology Society [10,17]. These activities were combined with the CARE-NMD professional survey to avoid duplication, with qualitative questions incorporated into the CTSR. The project resulted in refreshed data from sites already registered, particularly in the UK where it was integrated with national network-building and audit activities, and new registrations by previously non-registered sites.

## Discussion

To the best of our knowledge, the CTSR is the largest and most comprehensive database of neuromuscular centres in the world. It reduces the need for researchers to collate their own datasets on neuromuscular centres, in turn preventing duplication and wasted effort. A key strength lies in the independence and inclusiveness of the database, as registration is open to any centre and the data is held for the benefit of the neuromuscular community rather than being proprietary to one organisation. The data currently held in the CTSR is not exhaustive, and further work is being carried out to improve geographical and disease coverage. A sizable cohort of patients does not attend specialist centres (Kirschner, CARE-NMD unpublished data), while in some countries a significant proportion of neuromuscular centres have not yet registered in the CTSR. For example, the number of DMD patients accounted for in the CTSR (373) attending French neuromuscular centres is much lower than reported in the national mutational database (3,373) [18]. This may be due in part to the low number of centres registered in the CTSR from France (compared, for example, to those reported in the Orphanet Expert Centres database). In contrast, the French UMD-DMD patient registry captures all patients with a mutation in the dystrophin gene detected by any accredited genetic laboratory in France. Therefore, patients with other forms of dystrophinopathies, patients not attending neuromuscular centres and even deceased patients are accounted for [19,20]. In other countries such as the UK, the number of DMD patients reported in the CTSR exceeds the self-report patient registry, and the coverage of sites in the CTSR is more exhaustive. CTSR data may therefore be supplemented by other sources, including national patient registries and other surveys. However, while acknowledging these limitations, we believe that CTSR is of significant value to researchers.

The structure of the database allows the selection of centres according the specific needs of any given project. When planning a clinical trial, inclusion and exclusion criteria (e.g. age, genetic mutation, steroid and/or ambulatory status) and willingness to take part restrict the number of patients that can be recruited at an individual site. For example, even the most common genetic mutation amenable to exon-skipping (skipping of exon 51) is only found in 13% of patients [21]. Considering these factors, and that usually at least 5 recruited patients per site are sought, trial organisers may wish to screen sites to those that see a certain minimum number of patients – for instance 50 – with the disease of interest.

Furthermore, the CTSR is flexible and extensible to serve unforeseen needs, as demonstrated by the additional utility added to the core database by project-specific additions for CARE-NMD. This project provides an example of how the CTSR platform may be extended and used for innovative research, providing benefits to the neuromuscular field beyond those initially envisaged. It also demonstrates how national collaborative efforts with patient organisations and professional societies can help ensure record accuracy. The CTSR can help to determine the level of care available, in addition to feasibility or clinical trial readiness. It is also adaptable to different national and cultural contexts, for example by displaying different questions based on a site’s country of registration. This survey reveals several important factors about neuromuscular centres and care provision in different countries. The overall CTSR cohort appears to be broadly representative of that described in previous studies. Patients with DMD represent the largest group of those in the under 18 cohort, whilst those with Myotonic Dystrophy (DM1) form the largest group of adults. In 2009 Norwood *et al.* studied prevalence of neuromuscular diseases in the Northern region in the UK, and found that the five largest categories of disease were DM1 (28.1%), B/DMD (~20% taken together), FSHD (10.7%), SMA(5.1%), and LGMD (6.15%) [22]. The overall population reported in the CTSR does not match this (17.0% DM1, 37.4% B/DMD, FSHD 9.02%, SMA 14.3%, LGMD 15.3%), tending to show a higher proportion of childhood-onset conditions and lower of adult-onset.

We believe this is due to a better representation of paediatric patients in the CTSR. This could be because adult patients are more likely to be seen by their family doctor or a neurologist, rather than attending a specialist neuromuscular centre. The better representation of children in the CTSR (as compared to adults) may also reflect the relative severity of childhood-onset conditions that might stimulate significant interest in clinical trial participation and registration. By contrast, there may be an under-diagnosis of mild or late adult-onset conditions which is reflected in lower CTSR registrations. With the exception of LGMD, the adult cohort of the CTSR shows far closer correspondence with Norwood’s data (DM1 27.8%, B/DMD 22.2%, FSHD 15.4%, SMA 8.40%, and LGMD 22.4%), which is comprehensive in its coverage of the Northern region of the UK.

However, a recurrent theme from these data is that the demographics and structure of individual neuromuscular centres, as opposed to the overall population, can vary significantly between different countries. The heterogeneous nature of neuromuscular sites is evident even between countries that might otherwise be considered similar such as the UK, Germany, and France, while centre size can vary significantly between countries with similarly sized populations. Taking DMD as a case study in the three countries with the most centres, the size of centres that report seeing these patients varies dramatically: an average of 96 patients per site in the US, 52 in the UK, and 25 in Germany. Although the US (and Australia, with an average of 142 patients per site) are perhaps special geographic cases, the UK and Germany are not dissimilar and population density would not seem to explain the difference in average numbers of DMD patients seen between these two countries. This suggests that the characteristics of sites are influenced by factors other than obvious demographic and geographic considerations, such as the structure of national health care systems. As registration in the CTSR is open to any institution, it is possible that in some countries sites have registered that are not neuromuscular reference centres but still see a small number of neuromuscular patients. As accreditation of neuromuscular centres is very different from country to country this would require more in depth analysis on a national level.

A large number of sites might improve geographic accessibility to clinical and/or trial expertise. However, it is also known that the quality of such expertise for rare conditions depends upon regular exposure, in turn implying a minimally viable number of patients seen at an individual centre. Furthermore, the additional overheads associated with conducting clinical trials across many centres with small patient populations may be prohibitive, and prevent their participation in research. For these reasons, we suggest that care is taken when planning networks of neuromuscular centres to strike a careful balance between a minimally viable centre size and sufficient provision for the population to be served.

Regarding the size of centres and their relation to care, it may be worth examining the practices of rare disease centres for other conditions such as Cystic Fibrosis (CF). For example, the 2011 UK CF Registry Annual Data Report lists 32 paediatric and 28 adult specialist centres (with some centres serving both populations) [19]. Although these too vary in size, it is immediately apparent that most sites are significantly larger than those serving neuromuscular conditions. Although this may be in part explained by the higher prevalence of CF, it provides an opportunity to investigate the link between centre size and care, for the primary purpose of the UK CF registry is to help drive up the standard of clinical care and a great deal of clinical data is collected in order to allow this. Whilst mindful of the differences between neuromuscular care requirements and those for CF, this may be a fruitful area for further investigation. Such an approach has recently been advocated for DMD patient registries by Scully *et al.*[23].

### Challenges

As with any self-report database, a significant challenge is in ensuring data accuracy. Site visits by study monitors would be an excellent but expensive way to ensure data accuracy, and are not feasible in the current setting with limited resources. Errors can arise from mistakes in data entry, though this is minimised by validation at the input stage. As time passes, inaccuracies can occur naturally as information, particularly with regards to patient cohort and site facilities, becomes outdated. This issue is mitigated in the CTSR via an update process whereby sites are contacted at least annually and asked to update their records. In addition, secondary checks are carried out upon receipt of individual enquiries: those sites within the scope of the enquiry are contacted to confirm that the data held for them is accurate.

This curation of data over time requires time and effort on the part of the CTSR managers and the sites themselves. However, the benefits to the neuromuscular field of a central source of up-to-date data on cohort and capabilities of a large number of sites outweigh the costs associated with maintaining this infrastructure. Furthermore, motivation for sites to participate and keep their data accurate is provided through the potential for them to be considered for proposed trials, and in recognition of their collaboration internationally.

### Future developments

The CTSR provides a flexible and extensible platform for the neuromuscular field, and a variety of further developments are possible. At the simplest these might be minor extensions with the creation of disease-, country- or study-specific modules to capture additional information. Similarly, more sophisticated access permissions could be created to enable specific categories of users (such as trial co-ordinators or national curators) with very specific abilities to view or edit particular types of data.

In the longer term, it might be possible to link existing patient registries to the CTSR, which would enable the correlation of health information with site information. This would provide valuable insights into links between health outcomes and the capabilities of neuromuscular sites. While there may be logistical and cultural obstacles to such linkage, it would enable the integration of patient and centre-level data which would potentially allow causal links to be identified.

The CTSR platform is also applicable to other disease areas. For example, within the EU-funded NeurOmics project the CTSR is currently being extended to cover a range of neurodegenerative diseases, which will allow the integration of a large number of new centres [24].

In addition, the CTSR might be used to capture defined criteria for national or international centres of expertise or for European Reference Networks for neuromuscular diseases.

## Conclusion

The TREAT-NMD Care and Trial Site Registry is an example of a field-specific online self-report database for neuromuscular sites. Although originally envisaged primarily as a tool for clinical trial readiness (as part of a wider package of translational research tools in the TREAT-NMD Network), it has proved valuable for purposes beyond those originally considered. The CTSR platform will continue to be developed by the TREAT-NMD Alliance. The utility of such a registry of sites has been demonstrated through numerous enquiries since its establishment in 2007, and other Rare Disease areas may wish to consider developing similar initiatives.

## Competing interests

All authors and affiliated institutions are involved in the TREAT-NMD Alliance. KB and VS led the TREAT-NMD Network of Excellence 1997–2011, and HL has chaired the TREAT-NMD Alliance since that date. SR is based in the TREAT-NMD Alliance Secretariat. JK leads the TREAT-NMD Clinical Trials Co-ordination Centre (CTCC) at University Medical Center Freiburg and is member of the TREAT-NMD Alliance executive committee. AT, KG, and KK are involved with running the CTSR.

## Authors’ contributions

JK, HL and KB conceived of the study. AT, KG, and KK provided data for the study from the CTSR. SR analysed the data, and drafted and edited the manuscript with guidance from HL, KB, VS and JK. All authors read and approved the final manuscript.

## Authors’ information

SR works at the TREAT-NMD Alliance Secretariat, Institute of Genetic Medicine, Newcastle University.

HL is Professor of Experimental Myology, Institute of Genetic Medicine, Newcastle University, and current chair of the TREAT-NMD Alliance Executive Committee.

AT and KK are IT Specialists and Consultants at University Medical Center, Freiburg.

KG is a Project Manager at University Medical Center, Freiburg.

KB is Action Research Chair of Neuromuscular Genetics, Institute of Genetic Medicine, Newcastle University.

VS is the Harold McMillan Professor of Medicine, Institute of Genetic Medicine, Newcastle University.

RK is Director, Neuropaediatrics and Muscular Disorders, University Medical Center, Freiburg.

JK is Co-ordinator of the CTSR, University Medical Center, Freiburg.
